# Clinical Comparative Evaluation of Two New-Generation Optical Biometers: Intrasession Repeatability and Agreement of Biometric Parameters

**DOI:** 10.3390/diagnostics16040526

**Published:** 2026-02-10

**Authors:** Farid J. Bedrán, David P. Piñero

**Affiliations:** 1 Group of Optics and Visual Perception, Department of Optics, Pharmacology and Anatomy, University of Alicante, 03690 Alicante, Spain; faridbega85@gmail.com; 2 Clínica de Oftalmología San Diego, Medellín 050016, Colombia; 3 Advanced Clinic Optometry Unit, Department of Ophthalmology, Vithas Medimar International, 03016 Alicante, Spain

**Keywords:** optical biometry, swept-source OCT, axial length, intraocular lens power calculation, myopia management

## Abstract

**Objectives:** This study aims to evaluate the intrasession repeatability of the HBM-1 (Huvitz) optical biometer and to assess its agreement with the IOLMaster 700 (Zeiss) for the main biometric parameters used in intraocular lens (IOL) power calculation and in myopia management. **Methods:** A cross-sectional observational study was conducted in 82 eyes of 82 patients with the following age distribution: pediatric 26.8%, young adults 35.4%, and older adults 37.8% (total range 6–79 years). Optical biometry was performed three consecutive times with both biometers in the same session by a single trained examiner. HBM-1 repeatability was assessed using the within-subject standard deviation (Sw), the repeatability coefficient, and the intraclass correlation coefficient (ICC). Agreement between biometers was analyzed using Bland–Altman plots (limits of agreement, LoA). **Results:** The HBM-1 showed excellent intrasession repeatability, with very low Sw values—on the order of hundredths of a millimeter for axial length (AL), anterior chamber depth (ACD), and lens thickness (LT), and hundredths of a diopter for keratometry—with ICC ≥ 0.97 for most parameters. The mean bias (HBM-1 vs. IOLMaster 700) was small: AL 0.012 ± 0.052 mm (*p* = 0.045; LoA: −0.09 to 0.11 mm), ACD 0.059 ± 0.068 mm (*p* < 0.001; −0.07 to 0.19 mm), LT 0.052 ± 0.090 mm (*p* < 0.001; −0.12 to 0.23 mm), and central corneal thickness 0.82 ± 7.12 μm (*p* = 0.301; −13.1 to 14.8 μm). For corneal diameter and corneal curvature, mean differences were small (≤0.07 D) and not statistically significant in most cases. Age was not associated with discrepancies in AL but showed weak correlations with some anterior segment differences, without clear clinical relevance. **Conclusions:** The HBM-1 demonstrated excellent intrasession repeatability and a good level of clinical agreement with the IOLMaster 700 in a broad population that included children, young adults, and older adults.

## 1. Introduction

High-precision ocular biometry constitutes a fundamental component of contemporary ophthalmology and optometry, as it allows for improved accuracy in intraocular lens (IOL) power calculation, facilitates the planning of personalized refractive strategies, and supports axial length monitoring in myopia management programs [1,2,3,4]. Optical biometers based on partial coherence interferometry and, more recently, swept-source optical coherence tomography (SS-OCT) have largely superseded ultrasound biometry, owing to their superior accuracy and reproducibility in the measurement of axial length and anterior segment parameters [2,5,6].

The IOLMaster 700 (Carl Zeiss Meditec, Jena, Germany), based on SS-OCT, has become an established clinical reference standard in ocular biometry, supported by robust evidence of its repeatability, reliability, and agreement with other optical biometers, including in cataract populations [6,7,8,9,10]. In parallel with advances in optical biometry, novel devices integrating biometry with additional functionalities for anterior segment analysis and/or myopia monitoring have been introduced, underscoring the need to benchmark their performance against consolidated standards using repeatability and agreement metrics [4,11,12].

The HBM-1 (Huvitz, Anyang, Republic of Korea) is an optical low coherence reflectometry (OLCR)-based optical biometer that provides axial length and key anterior segment parameters. It integrates pupillometry and corneal analysis features for routine biometry workflows. However, published evidence on this device is very limited, and to the best of our knowledge, no paired study has directly compared it with the IOLMaster 700.

In addition, interest in axial length monitoring in children has increased markedly with the expansion of myopia management strategies and the need to detect small axial changes with high reliability [4,12,13,14]. Recent studies have shown that different optical biometers can be used in pediatric populations with good repeatability, but also that inter-device biases may exist, discouraging alternating devices during longitudinal follow-up [14,15]. Validating a general-purpose biometer such as the HBM-1 in a cohort that includes children allows its indication to be extended to myopia management programs, enabling the use of a single device for both phacorefractive surgery planning and refractive follow-up.

The aim of this study was to compare the intrasession repeatability of the HBM-1 and its agreement with the IOLMaster 700 for the main biometric parameters (AL, ACD, LT, CCT, WTW, and keratometry) in a mixed-age cohort assessed in a real-world clinical practice setting, including patients enrolled in myopia management programs.

## 2. Methods

### 2.1. Study Design and Participants

A cross-sectional observational study was conducted in a private optometry practice located within the facilities of Ophthalmology Clinic San Diego in Medellín (Colombia). The practice is independently operated and is not institutionally affiliated with the clinic. Eighty-two eyes from 82 consecutive patients requiring optical biometry for cataract surgery planning, pre- or postoperative refractive evaluation, or myopia follow-up were included. Inclusion criteria were sufficient ocular media clarity to allow AL acquisition using SS-OCT or OLCR, adequate fixation, and age of 5 years or older. Eyes were excluded if they had prior intraocular surgery (other than uncomplicated cataract surgery in the fellow eye), advanced corneal pathology, retinal pathology compromising fixation, or repeatedly poor measurement quality with either device.

For patients who had both eyes measured, only one eye (the right eye) was included to avoid statistical dependence. The study adhered to the principles of the Declaration of Helsinki, and all participants, or their legal guardians in the case of minors, provided written informed consent.

Each patient was informed about the characteristics and justification of the present study. All patients signed an informed consent form following the tenets of the Declaration of Helsinki. This research was approved by the Ethics Committee of the University of Alicante (No. UA-2021-01-14, and date of approval 14 January 2021).

### 2.2. Optical Biometers

The HBM-1 (Huvitz, Anyang, Republic of Korea) is an optical biometer based on optical low-coherence reflectometry (OLCR) that provides measurements of AL, ACD, LT, CCT, WTW, and dynamic pupillometry, fully integrated with a Placido-disc corneal topography system that provides corneal aberrometry and axial and tangential multi-annular keratometry. It includes automated alignment, internal fixation, and rapid acquisition of multiple examinations along the visual axis.

The IOLMaster 700 (Carl Zeiss Meditec, Jena, Germany) is a widely validated SS-OCT biometer that measures AL, ACD, LT, CCT, pupillometry, keratometry, total keratometry, and other anterior segment parameters and integrates with modern formulas for IOL power calculation [6,7,8,9,10]. In this study, it was used as the reference to evaluate the clinical agreement of the HBM-1.

### 2.3. Measurement Protocol

Following a comprehensive optometric and ophthalmological examination, biometric examinations were performed in separate rooms under controlled ambient illumination by a single experienced examiner, adhering to a standardized sequence and each device’s user manual. For each patient, three consecutive measurements were obtained with the HBM-1 and three with the IOLMaster 700. In approximately half of the cases, the device order was reversed to minimize order effects. Adequate blinking was ensured, and any measurement that did not meet a device’s built-in quality criteria was repeated. For inter-biometer agreement analysis, the average of the three measurements from each device was used. Axial length acquisition was successful in all included eyes with both devices (82/82; 100%).

### 2.4. Study Variables

The following parameters were analyzed: AL (mm), ACD (mm), LT (mm), CCT (µm), WTW (mm), flat and steep keratometry (Kf and Ks, D), mean keratometry (Avg K, D), and keratometric cylinder (Cyl, D). The astigmatism was further expressed using the vector components J_0_ and J_45_ (D). For consistency, all CCT values from the HBM-1 (reported in millimeters) were converted to micrometers (mm × 1000) to enable direct comparison with the IOLMaster 700 outputs. The J_0_ and J_45_ components were derived from cylinder power and axis using the Thibos vector transformation, which facilitates conventional parametric statistical analysis [16].

### 2.5. Statistical Analysis

Statistical analyses were performed using SPSS version 23 (IBM Corp., Armonk, NY, USA). The sample’s age was described using the mean, standard deviation, median, range, and 95% confidence interval. The intrasession repeatability of the HBM-1 was evaluated for each parameter using repeated-measures analysis of variance (ANOVA). From this model, the within-subject standard deviation (Sw) was derived as the square root of the within-subject error mean square (MSwithin) [6,11,17]. The repeatability coefficient (CR = 2.77 × Sw) was then calculated, which estimates the interval within which 95% of the differences between two repeated measurements of the same eye are expected to lie. Furthermore, the intraclass correlation coefficient (ICC) was computed using a two-way mixed-effects model for absolute agreement to quantify measurement reliability [6,11,18].

For comparisons between the HBM-1 and IOLMaster 700, the difference HBM-1 − IOLMaster 700 was calculated for each parameter and analyzed using a paired Student’s *t* test. Bland–Altman plots were constructed to visualize agreement and to estimate the mean bias with 95% limits of agreement (mean ± 1.96 × SD) [5,11,19,20,21]. Additionally, Pearson’s correlation coefficient was calculated to assess the relationship between patient age and each inter-device difference. A two-sided *p* value < 0.05 was defined as the threshold for statistical significance.

### 2.6. Sample Size and Power Calculation

An a priori sample size calculation was performed using G*Power software (version 3.1) for a two-tailed paired *t*-test (difference between two dependent means). The inter-device difference in AL was prespecified as the primary outcome for this estimation because AL is critical for IOL power calculation and myopia monitoring. Assuming a moderate standardized effect size (Cohen’s dz) of 0.5 (dz = Δ/SDdiff), which corresponds to detecting an inter-device AL bias of approximately 0.02 mm with an assumed standard deviation (SD) of the paired differences of 0.04 mm, a sample of 54 paired eyes was required for α = 0.05 (two-sided) and 95% power. The final sample included 82 paired eyes, exceeding this a priori requirement. A post hoc power calculation confirmed an achieved power of 0.994 for *n* = 82 under the same assumptions. Because standard sample size formulas do not directly apply to ICC and Bland–Altman agreement analyses, these metrics were not used for the a priori calculation; thus, any related subgroup analyses are considered exploratory.

## 3. Results

### 3.1. Sample Characteristics

Eighty-two cases were included, with no missing data for age. Age group distribution was: pediatric 22 (26.8%), young adult 29 (35.4%), and older adult 31 (37.8%). Mean ages of these subgroups ages (mean ± SD; min–max) were: pediatric 11.64 ± 3.84 (6–17), young adult 24.52 ± 6.67 (18–39), and older adult 61.65 ± 9.52 (41–79) (Table 1). Overall, the cohort covered a broad age range (6–79 years), consistent with a real-world clinical practice population.

### 3.2. HBM-1 Intrasession Repeatability

Repeated-measures analyses demonstrated excellent intrasession repeatability of the HBM-1, with very low within-subject variability for all parameters (Table 2). Sw values were in the hundredths of a millimeter for AL, ACD, and LT; approximately 10–15 μm for CCT; and in the tenths of a diopter for keratometry. ICCs for individual measurements exceeded 0.95 for all parameters, ranging from 0.97 to 0.99 for AL, ACD, LT and CCT.

Table 2 summarizes the HBM-1 intrasession repeatability indices (Sw, CR, and ICC for each variable).

### 3.3. Differences Between HBM-1 and IOLMaster 700

**Figure 1:**
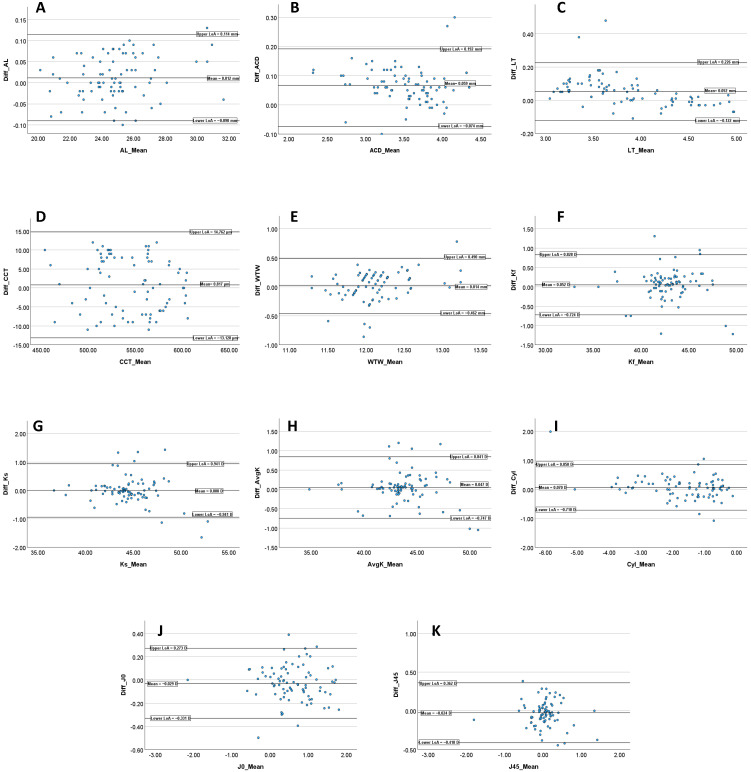
Bland–Altman plots of the agreement between the HBM-1 and the IOLMaster 700 for the different biometric parameters: AL, axial length (**A**); ACD, anterior chamber depth (**B**); LT, lens thickness (**C**); CCT, central corneal thickness (**D**); WTW, white-to-white corneal diameter (**E**); K1, flat keratometry (Kf) (**F**); K2, steep keratometry (Ks) (**G**); Avg K, mean keratometry (**H**); Cyl, corneal cylinder (**I**); J_0_ (**J**) and J_45_ (**K**). The central horizontal line represents the mean difference (bias) and the upper and lower lines represent the 95% limits of agreement; values are annotated on each panel.

The mean values, standard deviations, mean inter-device difference (Δ = HBM-1 − IOLMaster 700), range of differences, and mean absolute difference for all parameters are presented in Table 3. Bland–Altman plots illustrating the agreement for each parameter are shown in Figure 1, with the corresponding mean bias and 95% limits of agreement. Paired-*t* tests revealed statistically significant inter-device mean differences for AL (*p* = 0.045), ACD (*p* < 0.001), and LT (*p* < 0.001). In contrast, no statistically significant differences were observed for CCT, WTW, corneal cylinder, mean keratometry, K1, K2, or the astigmatism vectors (J_0_ and J_45_) (Table 3).

### 3.4. Correlation Between Age and Inter-Device Differences

**Figure 2:**
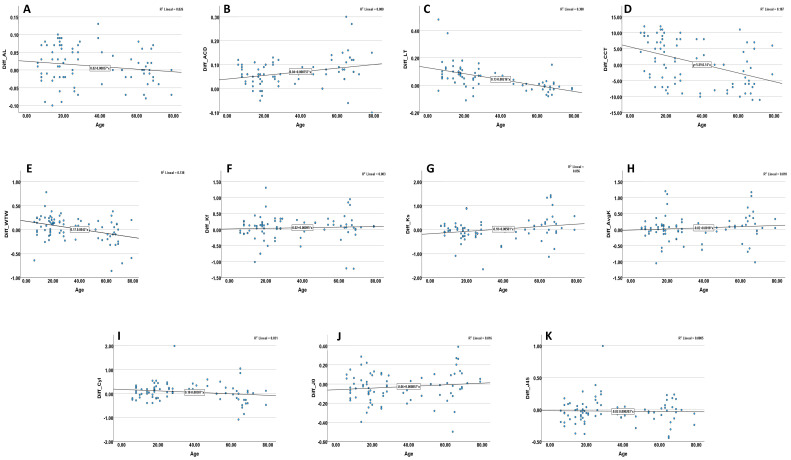
Relationship between age and inter-device differences (HBM-IOL) for the different biometric parameters: AL, axial length (**A**); ACD, anterior chamber depth (**B**); LT, lens thickness (**C**); CCT, central corneal thickness (**D**); WTW, white-to-white corneal diameter (**E**); K1, flat keratometry (Kf) (**F**); K2, steep keratometry (Ks) (**G**); Avg K, mean keratometry (**H**); Cyl, corneal cylinder (**I**); J_0_ (**J**) and J_45_ (**K**). Each panel represents a specific parameter, with the fitted linear regression line and coefficient of determination (R^2^).

Pearson correlations between patient age and inter-device differences for each biometric parameter are shown in Table 4 and visually in Figure 2. Age was not significantly associated with differences in axial length (Diff_AL: r = −0.162; *p* = 0.145; Figure 2A), keratometric cylinder (Diff_Cyl: r = −0.175; *p* = 0.116; Figure 2I), Avg K (Diff_AvgK: r = 0.101; *p* = 0.368; Figure 2H), or the astigmatism vector component J_0_ and J_45_ (Diff_J_0_: r = 0.126; *p* = 0.259; Figure 2J; Diff_J_45_: r = 0.023; *p* = 0.835; Figure 2K). A weak positive correlation was observed between age and the ACD difference (Diff_ACD: r = 0.284; *p* = 0.010; Figure 2B). Conversely, age demonstrated moderate negative correlations with differences in LT, CCT, and WTW diameter (Diff_LT: r = −0.547; *p* < 0.001; Figure 2C; Diff_CCT: r = −0.432; *p* < 0.001; Figure 2D; Diff_WTW: r = −0.371; *p* = 0.001; Figure 2E). For keratometry, a weak positive correlation was found for K1 (flat) (Diff_K1: r = 0.236; *p* = 0.033; Figure 2F), while no significant association was observed for K2 (steep) (Diff_K2: r = 0.054; *p* = 0.628; Figure 2G).

## 4. Discussion

### 4.1. Main Interpretation

This study demonstrates that the HBM-1 exhibits excellent intrasession repeatability and high agreement with the IOLMaster 700 in a cohort representative of clinical practice, including a wide age range (6–79 years), supporting the clinical applicability of the findings in both pediatric and adult populations; however, age subgroup results should be interpreted in light of the sample size within each category. Mean bias for axial length was only 0.0116 mm and limits of agreement were approximately ±0.10 mm, values considered clinically acceptable for IOL power calculation and axial length monitoring [4,5,6,8,11,14,21].

Importantly, a small mean bias at the cohort level can mask larger individual discrepancies. The range of observed differences and the mean absolute difference (|Δ|) presented in Table 3 provide a more informative view of dispersion and help identify parameters with sporadic outliers (e.g., ACD differences up to 0.30 mm, Cyl up to 1.99 D, and CCT up to 12 µm). In older adults, greater variability may be attributable to factors such as reduced optical quality, tear film instability, fixation variability, or lens opacity. Consequently, direct interchangeability of the two devices should be approached with caution, particularly when measurements fall near clinical decision thresholds, even in the context of negligible overall mean bias.

The statistically significant inter-device differences observed for ACD and LT, while methodologically relevant, are unlikely to be clinically significant for IOL power calculation when using fifth-generation formulas [3], especially if the same device is used consistently for preoperative planning. However, in eyes with extreme biometric values or in candidates for premium IOLs, it would be prudent to verify the consistency of biometric data and consider employing multiple formulas if substantial inter-device discrepancies arise [3,22,23].

As shown in Table 4 and Figure 2, inter-device differences were not age-invariant for all parameters. Moderate negative correlations were found between age and the differences for LT (r = −0.547, *p* < 0.001), CCT (r = −0.432, *p* < 0.001), and WTW (r = −0.371, *p* = 0.001), indicating a tendency for the HBM-1 to yield progressively lower values relative to the IOLMaster 700 in older individuals. Clinically, this age-dependent variation suggests that device interchangeability is not uniform across the lifespan, particularly for parameters reliant on boundary detection or image contrast (e.g., LT, WTW). Potential contributors include age-related changes in crystalline lens morphology affecting LT segmentation, reduced limbal contrast influencing WTW estimation, and inherent algorithmic differences in pachymetry measurements. Therefore, even with modest cohort-level mean differences, clinicians should avoid alternating devices when serially monitoring age-sensitive parameters, especially in older adults or when measurements approach critical clinical thresholds.

### 4.2. Comparison with Literature

Our results are consistent with the broader literature comparing SS-OCT, PCI, and OLCR biometers, which typically report small, clinically acceptable AL biases and narrow limits of agreement [5,6,7,8,18,19,20,24]. More recent large-scale and multi-device comparative studies have confirmed similar agreement for corneal and anterior segment parameters, although somewhat larger discrepancies have been noted for variables such as WTW or corneal cylinder, particularly when measurements involve different keratometry technologies or segmentation algorithms [11,21,25,26,27,28].

The high repeatability (low within-session variability and high ICCs) of SS-OCT biometers is well-established in both adult and cataract populations [9,10,17]. In pediatric cohorts, studies indicate good feasibility and repeatability but emphasize the importance of using the same device for longitudinal myopia monitoring [4,12,13,14]. Our findings contribute to this literature by demonstrating that a widely available clinical biometer, theHBM-1, achieves comparable levels of repeatability and agreement with the IOLMaster 700, even within a pediatric subgroup.

### 4.3. Implications for IOL Power Calculation

In IOL power calculation, a 0.1 mm difference in AL can induce a refractive error of approximately 0.25–0.30 D in eyes of average length [1,3]. Consequently, it is clinically desirable for the 95% limits of agreement between biometers to remain within ±0.10 mm. Our results confirm that the HBM-1 meets this criterion against the IOLMaster 700, supporting its use for planning both monofocal and premium IOLs. While the observed differences in ACD and LT are small, they could have a greater impact in formulas that rely heavily on effective lens position (ELP) estimation, particularly in eyes with extreme biometrics. In such cases, cross-checking calculations with multiple formulas is advisable [3].

### 4.4. Implications for Myopia Control

The cross-sectional agreement in AL between the HBM-1 and IOLMaster 700, coupled with its excellent intrasession repeatability in pediatric eyes, supports the potential of the HBM-1 for AL monitoring in myopia management, provided the same device is used for follow-up [4,13,14]. However, as this was a single-visit study, our findings establish device comparability at a single timepoint rather than validating the tracking of axial elongation over time. Prospective longitudinal studies with repeated measurements over intervals relevant to myopia progression (e.g., 6–12 months) are required to confirm inter-device agreement for change in AL over time and to define the smallest detectable change for myopia control applications [12]. Until such longitudinal validation is available, alternating devices during AL follow-up for the same patient is not recommended, in order to avoid interpreting device-related changes as true progression [4,12,14,15].

### 4.5. Strengths and Limitations

The strengths of this study include its rigorous methodology and broad clinical applicability. Key features are the acquisition of repeated measurements per device, the inclusion of a wide age spectrum (6–79 years) encompassing a pediatric subgroup, and the comprehensive evaluation of biometric parameters relevant to both cataract surgery and myopia management. Furthermore, the analysis incorporated robust assessments of intrasession repeatability (Sw, CR, ICC) and inter-device agreement (paired *t*-tests, Bland–Altman analysis, and correlation with patient age) [1,3,4,6,11]. This study has several limitations. First, its cross-sectional design precludes longitudinal assessment of AL change. Second, while the sample size is moderate and comparable to other biometer validation studies, the single-center setting may limit the generalizability of the findings. Third, although the cohort was stratified by age, other clinically relevant covariates—such as refractive status, extreme axial lengths (e.g., <22 mm or >26 mm), and lens opacity density—were not prespecified for subgroup analysis. Future studies should incorporate these variables, ideally including standardized lens opacity grading (e.g., LOCS III) and device signal-quality metrics, and should be adequately powered to evaluate extreme-eye subgroups.

It is also important to note that the study was not statistically powered for stratified comparisons. Consequently, subgroup analyses involving small samples should be interpreted with caution. The precision of agreement metrics (e.g., Bland–Altman limits of agreement, ICC confidence intervals) is dependent on sample size and measurement variability. For the overall cohort (*n* = 82), the observed SD of AL differences (0.0516 mm) yields a 95% confidence interval half-width of approximately 0.02 mm around each limit of agreement endpoint (calculated as 1.96 × √3 × SDdiff/√*n*), indicating reasonably precise estimates for AL agreement. However, precision is reduced for parameters with greater variability and within smaller subgroups.

## 5. Conclusions

The HBM-1 optical biometer demonstrated excellent intrasession repeatability and strong clinical agreement with the IOLMaster 700 for axial length and key anterior segment parameters in a cohort spanning adults and children. Mean inter-device differences were small, and the 95% limits of agreement for axial length fell within the clinically acceptable range for modern IOL power calculation and for cross-sectional monitoring in myopia management. While these findings support the use of the HBM-1 as a reliable alternative to the IOLMaster 700 in routine clinical practice, consistent use of the same device for individual patient follow-up is essential. Future studies are needed to confirm device interchangeability in eyes with extreme biometrics and to validate its performance for tracking axial elongation over time.

## Figures and Tables

**Table 1:** Sex and age-group distribution. Abbreviations: SD, standard deviation; IQR, interquartile range; CI, confidence interval.

Age Group	*n* (%)	Age, Mean ± SD (Min–Max)	Median (IQR)	95% CI of the Mean
Pediatric	22 (26.8%)	11.64 ± 3.84 (6–17)	11.00 (7)	9.94–13.34
Young adult	29 (35.4%)	24.52 ± 6.67 (18–39)	25.00 (9)	21.98–27.06
Older adult	31 (37.8%)	61.65 ± 9.52 (41–79)	64.00 (14)	58.15–65.14
Eye analyzed	One eye per patient			
Female sex, *n* (%)	46 (56.1%)			
Male sex, *n* (%)	36 (43.9%)			
Total	82 (100%)			

**Table 2:** HBM-1 intrasession repeatability indices. Abbreviations: Sw, within-subject standard deviation; CoV, coefficient of variation; ICC, intraclass correlation coefficient; D, diopters; CI, confidence interval; AL, axial length; ACD, anterior chamber depth; LT, lens thickness; CCT, central corneal thickness; WTW, white-to-white corneal diameter; Kf and Ks, flat and steep keratometry; Avg K, mean keratometry; Cyl, keratometric cylinder; J_0_ and J_45_, astigmatism vector components.

Parameter	Unit	HBM-1 Mean	Sw	CoV (%)	Repeatability (±2.77 × Sw)	ICC (Single Measure), 95% CI
AL	mm	24.8918	0.063	0.25	±0.17	0.999 (0.997–0.999)
ACD	mm	3.5573	0.009	0.25	±0.03	0.999 (0.999–1.000)
LT	mm	3.8855	0.012	0.31	±0.03	0.999 (0.998–1.000)
CCT	µm	545.37	1.430	0.26	±3.96	0.998 (0.995–0.999)
WTW	mm	12.1435	0.032	0.26	±0.09	0.993 (0.984–0.996)
Cyl	D	−1.6432	0.005	0.31	±0.01	1.000 (≈1.000–1.000)
Avg K	D	43.6867	0.118	0.27	±0.33	0.997 (0.993–0.998)
Kf (K1)	D	42.8856	0.118	0.28	±0.33	0.997 (0.994–0.998)
Ks (K2)	D	44.4880	0.120	0.27	±0.33	0.997 (0.994–0.998)
J_0_	D	0.5789	0.032	— *	±0.09	0.997 (0.994–0.998)
J_45_	D	−0.0016	0.071	— *	±0.20	0.974 (0.955–0.985)

* CoV is not reported for J_0_/J_45_ because these are vector components with a mean potentially close to zero and positive/negative sign, making the CoV poorly interpretable.

**Table 3:** Comparison between HBM-1 and IOLMaster 700 (*n* = 82 pairs). Values are reported as mean ± SD. Δ = HBM-1 − IOLMaster 700. Range of Δ corresponds to the observed minimum and maximum inter-device differences. |Δ| is the mean absolute difference. Paired-samples *t* test was used. CI, confidence interval.

Parameter	Unit	HBM-1 (Mean ± SD)	IOLMaster 700 (Mean ± SD)	Δ (Mean ± SD)	Range of Δ (Min to Max)	|Δ| (Mean ± SD)	95% CI for Δ	t	df	*p*	r
AL	mm	24.8918 ± 2.29622	24.8802 ± 2.28493	0.01159 ± 0.05158	−0.10 to 0.13	0.0421 ± 0.03169	0.00025 to 0.02292	2.034	81	0.045	1.000
ACD	mm	3.5573 ± 0.43531	3.4980 ± 0.45136	0.05927 ± 0.06769	−0.10 to 0.30	0.0734 ± 0.05179	0.04439 to 0.07414	7.929	81	<0.001	0.989
LT	mm	3.8855 ± 0.50285	3.8340 ± 0.54898	0.05146 ± 0.08982	−0.11 to 0.48	0.0720 ± 0.07421	0.03173 to 0.07120	5.189	81	<0.001	0.989
CCT	µm	545.37 ± 36.876	544.55 ± 37.757	0.817 ± 7.115	−11 to 12	6.33 ± 3.28	−0.746 to 2.380	1.040	81	0.301	0.982
WTW	mm	12.1435 ± 0.45609	12.1293 ± 0.40077	0.01427 ± 0.24334	−0.86 to 0.78	0.1935 ± 0.16856	−0.03920 to 0.06774	0.531	81	0.597	0.846
Cyl	D	−1.6432 ± 1.09891	−1.7127 ± 1.22728	0.06951 ± 0.39776	−1.08 to 1.99	0.2734 ± 0.29568	−0.01789 to 0.15691	1.583	81	0.117	0.947
Avg K	D	43.6867 ± 2.54609	43.6401 ± 2.58907	0.04659 ± 0.40451	−1.05 to 1.20	0.2661 ± 0.30683	−0.04230 to 0.13547	1.043	81	0.300	0.988
K1	D	42.8856 ± 2.59744	42.8333 ± 2.60803	0.05232 ± 0.39573	−1.22 to 1.31	0.2706 ± 0.29196	−0.03463 to 0.13927	1.197	81	0.235	0.988
K2	D	44.5288 ± 2.61514	44.5283 ± 2.72344	0.00049 ± 0.47964	−1.65 to 1.43	0.3071 ± 0.36687	−0.10490 to 0.10588	0.009	81	0.993	0.985
J_0_	D	0.5789 ± 0.64770	0.6081 ± 0.65497	−0.02918 ± 0.15366	−0.50 to 0.39	0.1192 ± 0.10046	−0.06294 to 0.00458	−1.720	81	0.089	0.972
J_45_	D	−0.0016 ± 0.47588	0.0221 ± 0.56136	−0.02370 ± 0.19655	−0.44 to 1.00	0.1350 ± 0.14407	−0.06689 to 0.01948	−1.092	81	0.278	0.941

**Table 4:** Pearson correlations between age and inter-device differences. Abbreviations: AL, axial length; ACD, anterior chamber depth; LT, lens thickness; CCT, central corneal thickness; WTW, white-to-white corneal diameter; Kf and Ks, flat and steep keratometry; Avg K, mean keratometry; Cyl, keratometric cylinder; J_0_ and J_45_, astigmatism vector components; D, diopters; r, coefficient of correlation.

Parameter (HBM − IOL Difference)	Unit	r (Age, Difference)	*p* (Two-Tailed)
Diff_AL	mm	−0.162	0.145
Diff_ACD	mm	0.284	0.010
Diff_LT	mm	−0.547	<0.001
Diff_CCT	µm	−0.432	<0.001
Diff_WTW	mm	−0.371	0.001
Diff_Cyl	D	−0.175	0.116
Diff_K1	D	0.054	0.628
Diff_K2	D	0.236	0.033
Diff_AvgK	D	0.101	0.368
Diff_J_0_	D	0.126	0.259

## Data Availability

The data that support the findings of this study are available from the corresponding author, DPP, upon reasonable request.
